# Associations of neutrophil to lymphocyte ratio and red cell distribution width on different days with the prognosis of aneurysmal subarachnoid hemorrhage patients

**DOI:** 10.1097/MD.0000000000036021

**Published:** 2023-11-24

**Authors:** Jie Min, Yongfeng Zhao, Xian Wang, Jian Zhao

**Affiliations:** a Neurointensive Care Unit, The First Affiliated Hospital of Yangtze University, Jingzhou, China; b Department of Hematology, The First Affiliated Hospital of Yangtze University, Jingzhou, China; c Department of Pharmacy, The First Affiliated Hospital of Yangtze University, Jingzhou, China.

**Keywords:** aneurysmal subarachnoid hemorrhage, lymphocyte, neutrophil, prognosis, red cell distribution width

## Abstract

The associations of neutrophil to lymphocyte ratio (NLR) and red cell distribution width (RDW) with the prognosis of aneurysmal subarachnoid hemorrhage (aSAH) patients were confirmed in a few studies. But NLR and RDW levels in most of these studies were on admission. Here we carried 1 retrospective study including 150 patients with aSAH who underwent surgeries in the First Affiliated Hospital of Yangtze University from January 2020 to February 2023 to explore the associations on the preoperative day, the first (1st), third (3rd), and seventh (7th) postoperative days. The level of RDW on the 3rd postoperative day and level of NLR on the 7th postoperative day in patients with poor prognosis were significantly higher than patients with good prognosis. The results of multivariate logistic analysis also confirmed the associations of RDW on the 3rd postoperative day (OR = 16.785, 95% CI: 4.077–69.107, *P < *.001) and NLR on the 7th postoperative day (OR = 8.399, 95% CI: 2.167–32.544, *P = *.002) with prognosis of aSAH patients. The results of receiver operating characteristic curve showed that cutoff values of RDW and NLR for predicting the prognosis in aSAH patients were 13.05% and 6.97, respectively. Higher RDW on the 3rd postoperative day and NLR on the 7th postoperative day were possibly associated with poor prognosis of aSAH patients. We should pay attention to the RDW and NLR levels during different hospitalization periods, especially in the short postoperative period. Moreover, the cutoff values for predicting prognosis need to be validated in larger-sample studies.

## 1. Introduction

Aneurysmal subarachnoid hemorrhage (aSAH) is a complex and fatal disease. Although breakthroughs have been made in the treatment of aSAH in recent years, the prognosis of some patients is still poor. The important roles of some inflammatory indicators were demonstrated in the progression of cerebrovascular diseases and possibly related to the prognosis in some studies.^[1–3]^ The Neutrophil to lymphocyte ratio (NLR), as one of the easily accessible biomarkers, has been a widely studied inflammatory indicator in recent years. Previous studies confirmed that NLR was associated with the occurrence, severity, and prognosis of some diseases. NLR was associated with the functional outcomes in patients with cerebral hemorrhage and acute cerebral infarction.^[4,5]^ On the other hand, thrombosis was also involved in the pathological changes after aSAH.^[6]^ Red cell distribution width (RDW) was a indicator reflecting the heterogeneity of red blood cell size and confirmed to be important and possibly associated with prognosis of patients in many diseases such as ischemic stroke,^[7]^ pulmonary embolism,^[8]^ and tumors.^[9]^

There were a few studies that explored the associations of NLR and RDW with the outcome of aSAH patients, and suggested that higher NLR and RDW were associated with poor prognosis.^[10,11]^ However, the NLR and RDW levels in most of these studies were on admission. The disease condition was variable during hospitalization. Therefore, we explored the associations on the preoperative day, 1st, 3rd and 7th postoperative days in this study.

## 2. Materials and methods

### 2.1. Patients

**Figure 1.**
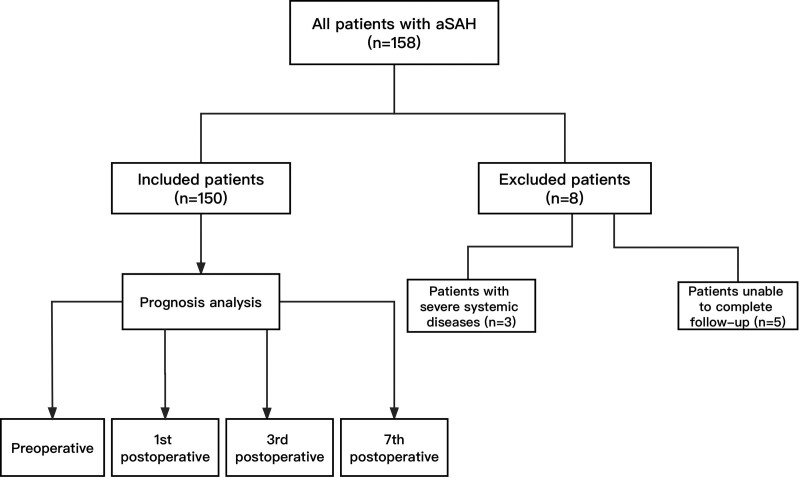
The flow chart of the study. Among the 150 patients included, there were 115 cases with good prognosis and 35 cases with poor prognosis.

We carried one retrospective study including 150 patients with aneurysmal subarachnoid hemorrhage who underwent surgeries in the First Affiliated Hospital of Yangtze University from January 2020 to February 2023. The study was approved by the Ethics Committee of the First Affiliated Hospital of Yangtze University. Patients meeting the following criteria were included: (1) had subarachnoid hemorrhage confirmed by computed tomography (CT); (2) had intracranial aneurysm confirmed by digital subtraction angiography (DSA) and associated with bleeding; (3) received craniotomy clipping or intravascular embolization surgeries after admission. Patients meeting the following criteria were excluded: (1) had subarachnoid hemorrhage caused by arteriovenous malformations and trauma (or did not have aneurysms); (2) suffered from severe systemic diseases, such as severe liver dysfunction, kidney dysfunction, heart dysfunction, hematological diseases, etc.; (3) had Modified Rankin Scale (mRS) score ≥ 3 before admission; (5) were unable to complete follow-up of 3 months. The flow chart was showed in Figure 1.

### 2.2. Data collection

Baseline characteristics included gender, age, Hunt-Hess grade, Glasgow Coma Scale (GCS) score, combination of intraventricular hemorrhage, the location of aneurysm, neck length of aneurysm, date of surgery, time length of surgery. The blood cells counts were extracted from the hospital information system including white blood cells, neutrophils, lymphocytes, hemoglobin, and the red blood cell distribution width on the preoperative day, the 1st, 3^rd^, and 7th postoperative days. NLR was the ratio of neutrophil to lymphocyte. The prognosis was determined by the Modified Rankin Scale (mRS) results of 90-d follow-up after morbidity. The patients with scores of mRS 0–2 were defined as having a good prognosis, while mRS 3–6 were defined as having a poor prognosis.

### 2.3. Statistical analysis

We used SPSS 23.0 (IBM SPSS Inc., Chicago, IL) for data analysis. If the quantitative variables met the normal distribution and variances were homogeneous, independent-sample *t* test was used for analysis. If the quantitative variables met the normal distribution but variances were not homogeneous, corrected *t* test was used. If the normal distribution could not be satisfied, Mann–Whitney *U* test was used for analysis. The qualitative variables were compared by Pearson chi-square test, Continuity correction or Fisher exact test. The multivariate logistic regression model was used for factors associated with prognosis. Covariates that had a *P*-value >.05 in the univariate analysis were added to the multivariate logistic analysis. ROC (receiver operating characteristic) curve was used for the cutoff values of characteristics for predicting poor prognosis in patients with aSAH. Graphpad prism 6.0 (GraphPad Software Inc., La Jolla, CA) was used for the correlation analysis. All *p*-values were 2-sided and the statistical significance was set at *P* < .05.

## 3. Results

### 3.1. Baseline characteristics

Among the 150 patients included, there were 57 males and 93 females. Among these patients, there were 50 cases with Hunt-Hess grade III–V and 26 cases with GCS score < 11. There were 71 cases combined with ventricular hemorrhage. The median aneurysm neck length was 3.50 mm. The proportions of Hunt-Hess grade III–V cases, cases with GCS score < 11 and with ventricular hemorrhage in patients with poor prognosis were 77.1%, 48.6%, and 80% respectively, which were significantly higher than patients with good prognosis, *P < *.05 (Table 1).

**Table 1:** Baseline clinical characteristics according to the prognosis of aSAH patients.

	All patients (n = 150)	Good prognosis (n = 115)	Poor prognosis (n = 35)	*P* value
Gender [n (%)]				
Male	57 (38.0)	40 (34.8)	17 (48.6)	.141
Female	93 (62.0)	75 (65.2)	18 (51.4)	
Age (yr)	59.17 ± 9.22	58.59 ± 9.60	61.09 ± 7.68	.162
Hunt-Hess Grade [n (%)]				
III–V	50 (33.3)	23 (20.0)	27 (77.1)	<.001
I–II	100 (66.7)	92 (80.0)	8 (22.9)	
GCS score [n (%)]				<.001
≥11	124 (82.7)	106 (92.2)	18 (51.4)	
<11	26 (17.3)	9 (7.8)	17 (48.6)	
Combination with intraventricular hemorrhage [n (%)]				
Yes	71 (47.3)	43 (37.4)	28 (80)	<.001
No	79 (52.7)	72 (62.6)	7 (20)	
Neck length of aneurysm (mm)	3.50 ± 1.63	3.48 ± 1.33	3.59 ± 3.07	.170
Location of aneurysm [n (%)]				
Internal carotid artery system	144 (96.0)	110 (95.7)	34 (97.1)	1.000
Vertebrobasilar artery system	6 (4.0)	5 (4.3)	1 (2.9)	
Daughter aneurysm [n (%)]				
Yes	87 (58.0)	70 (60.9)	17 (48.6)	.197
No	63 (42.0)	45 (39.1)	18 (51.4)	
Multiple aneurysms [n (%)]				
Yes	43 (28.7)	37 (32.2)	6 (17.1)	.085
No	107 (71.3)	78 (67.8)	29 (82.9)	
Time length of surgery (min)	180 ± 93	165 ± 75	180 ± 95	.092
Date of surgery (d)	2 ± 1	2 ± 1	2 ± 1	.690

aSAH = aneurysmal subarachnoid hemorrhage, GCS = Glasgow Coma Scale.

### 3.2. Associations of NLR and RDW on the preoperative day with the prognosis of aSAH patients

The median levels of NLR and RDW were 10.92% and 12.70% on the preoperative day in all patients. The preoperative white blood cells in patients with good prognosis was (10.97 ± 4.88) × 10^9^/L, significantly lower than patients with poor prognosis ([13.58 ± 8.26] × 10^9^/L), *P = *.001. The preoperative counts of neutrophils in patients with good prognosis and poor prognosis were (9.96 ± 4.00) × 10^9^/L and (12.77 ± 5.16) × 10^9^/L respectively, with significant difference, *P = *.001. The preoperative counts of lymphocytes had no significant difference, *P = *.379. The preoperative NLR in patients with good prognosis and poor prognosis were 10.69 ± 9.41 and 11.50 ± 10.25 respectively, with no significant difference (*P = *.461). The median preoperative RDW in patients with good prognosis was 12.7%, significantly lower than patients with poor prognosis (12.9%), *P = *.017. There was no significant difference in preoperative hemoglobin between patients with good prognosis and poor prognosis, *P = *.356 (Table 2).

**Table 2:** Associations of blood cell counts and other indicators with the prognosis of aSAH patients.

	All patients	Good prognosis (n = 115)	Poor prognosis (n = 35)	*P* value
On the preoperative day				
White blood cells (×10^9^/L)	11.51 ± 5.45	10.97 ± 4.88	13.58 ± 8.26	.001
Neutrophils (×10^9^/L)	10.62 ± 4.44	9.96 ± 4.00	12.77 ± 5.16	.001
Hemoglobin (g/L)	129.88 ± 15.88	129.00 ± 16.00	131.00 ± 31.00	.356
RDW (%)	12.70 ± 1.10	12.70 ± 1.20	12.90 ± 1.20	.017
Lymphocytes (×10^9^/L)	0.87 ± 0.55	0.87 ± 0.52	0.94 ± 1.02	.379
NLR	10.92 ± 9.29	10.69 ± 9.41	11.50 ± 10.25	.461
On the 1st postoperative day				
White blood cells (×10^9^/L)	10.50 ± 4.23	10.35 ± 3.70	12.57 ± 5.70	.001
Neutrophils (×10^9^/L)	8.91 ± 4.24	8.72 ± 3.63	11.14 ± 5.58	.001
Hemoglobin (g/L)	113.46 ± 15.70	113.95 ± 14.46	111.80 ± 19.47	.553
RDW (%)	13.00 ± 1.30	12.90 ± 1.30	13.25 ± 1.40	.001
Lymphocytes (×10^9^/L)	0.87 ± 0.49	0.95 ± 0.39	0.83 ± 0.37	.122
NLR	10.31 ± 7.67	9.86 ± 6.32	14.37 ± 13.05	.001
On the 3rd postoperative day				
White blood cells (×10^9^/L)	9.25 ± 4.04	8.85 ± 3.67	11.54 ± 4.40	<.001
Neutrophils (×10^9^/L)	7.50 ± 3.79	7.47 ± 2.79	9.78 ± 3.11	<.001
Hemoglobin (g/L)	117.01 ± 17.99	117.50 ± 24.00	108.50 ± 33.00	.063
RDW (%)	12.60 ± 1.30	12.40 ± 1.20	13.25 ± 1.70	<.001
Lymphocytes (×10^9^/L)	1.03 ± 0.67	1.05 ± 0.68	0.79 ± 0.64	.013
NLR	7.53 ± 5.58	6.46 ± 5.03	10.30 ± 7.16	<.001
On the 7th postoperative day				
White blood cells (×10^9^/L)	10.05 ± 3.43	9.38 ± 2.88	12.54 ± 4.16	<.001
Neutrophils (×10^9^/L)	7.88 ± 3.24	7.21 ± 2.67	10.27 ± 3.95	<.001
Hemoglobin (g/L)	118.70 ± 16.75	119.49 ± 15.56	115.73 ± 20.66	.367
RDW (%)	12.60 ± 1.23	12.40 ± 1.10	13.20 ± 1.25	<.001
Lymphocytes (×10^9^/L)	1.22 ± 0.62	1.26 ± 0.62	1.09 ± 0.59	.033
NLR	5.82 ± 4.54	5.63 ± 3.61	9.12 ± 9.62	<.001

aSAH = aneurysmal subarachnoid hemorrhage, NLR = neutrophil to lymphocyte ratio, RDW = red cell distribution width.

### 3.3. Associations of NLR and RDW on the 1st postoperative day with the prognosis of aSAH patients

On the 1st postoperative day, the median levels of NLR and RDW were 10.31 and 13.0% in all patients. The white blood cells in patients with good prognosis was (10.35 ± 3.70) × 10^9^/L, significantly lower than patients with poor prognosis ([12.57 ± 5.70] × 10^9^/L), *P = *.001. The counts of neutrophils in patients with good prognosis and poor prognosis were (8.72 ± 3.63) × 10^9^/L and (11.14 ± 5.58) × 10^9^/L respectively, also with significant difference, *P = *.001. The counts of lymphocytes in patients with good prognosis and poor prognosis were (0.95 ± 0.39) × 10^9^/L and (0.83 ± 0.37) × 10^9^/L, with no significant difference, *P = *.122. The NLR ratio on the 1st postoperative day in patients with good prognosis was 9.86 ± 6.32, significantly lower than patients with poor prognosis (14.37 ± 13.05), *P = *.001. The median RDW level in patients with good prognosis was 12.90%, significantly lower than that patients with poor prognosis (13.25%), *P = *.001. The levels of hemoglobin (*P = *.553) on the 1st postoperative day in patients with good prognosis and poor prognosis had no significant difference (Table 2).

### 3.4. Associations of NLR and RDW on the 3rd postoperative day with the prognosis of aSAH patients

On the 3rd postoperative day, the median levels of NLR and RDW were 7.53% and 12.60%. The white blood cells in patients with good prognosis was (8.85 ± 3.67) × 10^9^/L, significantly lower than patients with poor prognosis ([11.54 ± 4.40] × 10^9^/L), *P < *.001. The counts of neutrophils in patients with good prognosis and poor prognosis were (7.47 ± 2.79) × 10^9^/L and (9.78 ± 3.11) × 10^9^/L respectively, with significant difference, *P < *.001. The counts of lymphocytes on the 3rd postoperative day were (1.05 ± 0.68) × 10^9^/L and (0.79 ± 0.64) × 10^9^/L respectively, with significant difference, *P = *.013. The median NLR level in patients with good prognosis was 6.46, significantly lower than patients with poor prognosis (10.30), *P < *.001. The median RDW in patients with good prognosis on the 3rd postoperative day was 12.40%, significantly lower than patients with poor prognosis (13.25%), *P < *.001. There was no significant difference in hemoglobin level between patients with good prognosis and poor prognosis, *P = *.063 (Table 2).

### 3.5. Associations of NLR and RDW on the 7th postoperative day with the prognosis of aSAH patients

On the 7th postoperative day, the median levels of NLR and RDW were 5.82 and 12.60%. The white blood cells in patients with good prognosis was (9.38 ± 2.88) × 10^9^/L, significantly lower than patients with poor prognosis ([12.54 ± 4.16] × 10^9^/L), *P* < .001. The counts of neutrophils in patients with good prognosis and poor prognosis were (7.21 ± 2.67) × 10^9^/L and (10.27 ± 3.95) × 10^9^/L respectively, with significant difference, *P* < .001. The counts of lymphocytes on the 7th postoperative day were (1.26 ± 0.62) × 10^9^/L and (1.09 ± 0.59) × 10^9^/L respectively, with significant difference, *P* = .033. The median NLR level in patients with good prognosis was 5.63, significantly lower than patients with poor prognosis (9.12), *P* < .001. The median RDW in patients with good prognosis on the 7th postoperative day was 12.40%, significantly lower than patients with poor prognosis (13.20%), *P* < .001. There was no significant difference in hemoglobin level between patients with good prognosis and poor prognosis, *P* = .367 (Table 2).

### 3.6. The prognosis differences among patients with different quartiles of RDW and NLR

As showed in Table 3, we also compared the prognosis among patients with different quartiles of RDW and NLR. In patients with poor prognosis, the proportion of cases with lower quartile of RDW on the preoperative day was significantly lower than cases with higher quartile of RDW (*P = *.008). On the 1st postoperative day, the proportions of cases with lower quartiles of RDW and NLR were significantly lower than cases with higher quartiles (*P* = .004). On the 3rd postoperative day, the proportions of cases with lower quartiles of RDW (*P* = .001) and NLR (*P* < .001) were significantly lower than cases with higher quartiles. On the 7th postoperative day, the proportions of cases with lower quartiles of RDW (*P* = .003) and NLR (*P* < .001) were significantly lower than cases with higher quartiles.

**Table 3:** The prognosis differences among patients with different quartiles of RDW and NLR.

	All patients (n = 150)	Good prognosis (n = 115)	Poor prognosis (n = 35)	*P* value
RDW on preoperative day [n (%)]				.008
1st quartile	35 (23.6%)	33 (29.2%)	2 (5.7%)	
2nd quartile	33 (22.3%)	21 (18.6%)	12 (34.3%)	
3rd quartile	34 (23.0%)	28 (24.8%)	6 (17.1%)	
4th quartile	46 (31.1%)	31 (27.4%)	15 (42.9%)	
NLR on preoperative day [n (%)]				.727
1st quartile	37 (25.0%)	30 (26.5%)	7 (20.0%)	
2nd quartile	37 (25.0%)	28 (24.8%)	9 (25.7%)	
3rd quartile	37 (25.0%)	29 (25.7%)	8 (22.9%)	
4th quartile	37 (25.0%)	26 (23.0%)	11 (31.4%)	
RDW on 1st postoperative day [n (%)]				.004
1st quartile	35 (24.0%)	34 (29.8%)	1 (3.1%)	
2nd quartile	33 (22.6%)	26 (22.8%)	7 (21.9%)	
3rd quartile	41 (28.1%)	31 (27.2%)	10 (31.3%)	
4th quartile	37 (25.3%)	23 (20.2%)	14 (43.8%)	
NLR on 1st postoperative day [n (%)]				.004
1st quartile	37 (24.8%)	34 (29.8%)	3 (8.6%)	
2nd quartile	36 (24.2%)	28 (24.6%)	8 (22.9%)	
3rd quartile	39 (26.2%)	31 (27.2%)	8 (22.9%)	
4th quartile	37 (24.8%)	21 (18.4%)	16 (45.7%)	
RDW on 3rd postoperative day [n (%)]				.001
1st quartile	24 (16.3%)	21 (18.6%)	3 (8.8%)	
2nd quartile	45 (30.6%)	40 (35.4%)	5 (14.7%)	
3rd quartile	30 (20.4%)	25 (22.1%)	5 (14.7%)	
4th quartile	48 (32.7%)	27 (23.9%)	21 (61.8%)	
NLR on 3rd postoperative day [n (%)]				<.001
1st quartile	35 (23.6%)	32 (28.1%)	3 (8.8%)	
2nd quartile	39 (26.4%)	36 (31.6%)	3 (8.8%)	
3rd quartile	37 (25.0%)	27 (23.7%)	10 (29.4%)	
4th quartile	37 (25.0%)	19 (16.7%)	18 (52.9%)	
RDW on 7th postoperative day [n (%)]				.003
1st quartile	34 (24.6%)	30 (27.5%)	4 (13.8%)	
2nd quartile	30 (21.7%)	28 (25.7%)	2 (6.9%)	
3rd quartile	37 (26.8%)	29 (26.6%)	8 (27.6%)	
4th quartile	37 (26.8%)	22 (20.2%)	15 (51.7%)	
NLR on 7th postoperative day [n (%)]				<.001
1st quartile	35 (25.0%)	32 (29.1%)	3 (10.0%)	
2nd quartile	34 (24.3%)	29 (26.2%)	5 (16.7%)	
3rd quartile	37 (26.4%)	32 (29.1%)	5 (16.7%)	
4th quartile	34 (24.3%)	17 (15.5%)	17 (56.7%)	

NLR = neutrophil to lymphocyte ratio, RDW = red cell distribution width.

### 3.7. The multivariate logistic analysis and ROC curve results

**Figure 2.**
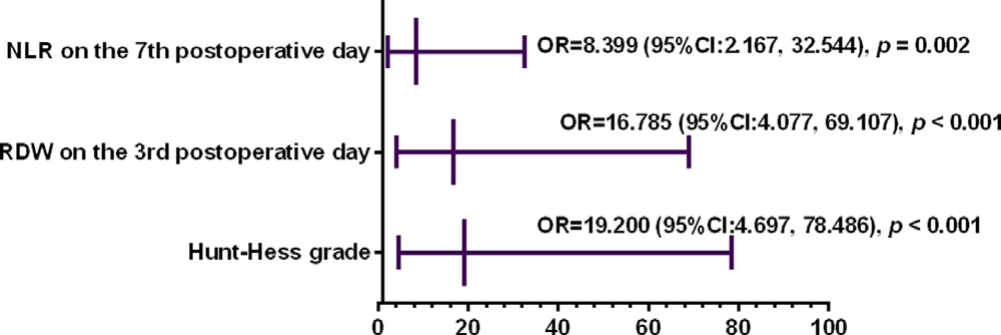
The results of multivariate logistic analysis. Hunt-Hess grade, RDW on the 3rd postoperative day and NLR on the 7th postoperative day were associated with the prognosis of aSAH patients, *P* < .05. aSAH = aneurysmal subarachnoid hemorrhage, RDW = red cell distribution width.

The results of multivariate logistic analysis showed that Hunt-Hess grade (OR = 19.200, 95% CI: 4.697–78.486, *P* < .001), RDW (OR = 16.785, 95% CI: 4.077–69.107, *P* < .001) on the 3rd postoperative day and NLR (OR = 8.399, 95% CI: 2.167–32.544, *P* = .002) on the 7th postoperative day were associated with the prognosis of aSAH patients (Fig. 2).

**Figure 3.**
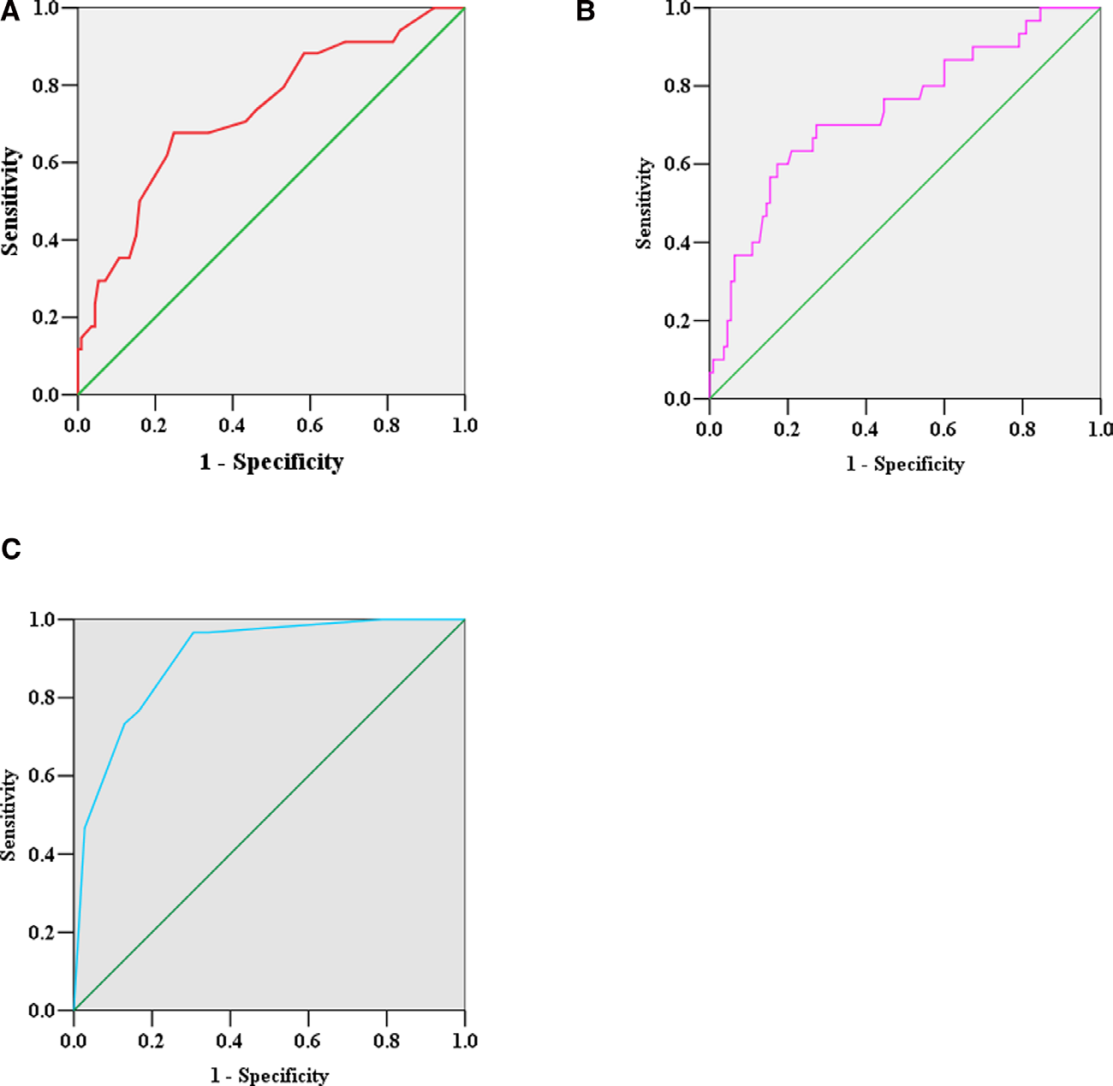
The ROC curves of RDW on the 3rd postoperative day (A), NLR on the 7th postoperative day (B), and multivariate logistic model (C). (A) The sensitivity and specificity of RDW ≥ 13.05% for predicting poor prognosis were 67.6% and 75.2%, respectively. (B) The sensitivity and specificity of NLR ≥ 6.97 for predicting poor prognosis were 41.2% and 89.9%, respectively. (C) The sensitivity and specificity of the multivariate logistic model for predicting poor prognosis were 73.7% and 90%, respectively. NLR = neutrophil to lymphocyte ratio, RDW = red cell distribution width, ROC = receiver operating characteristic.

**Figure 4.**
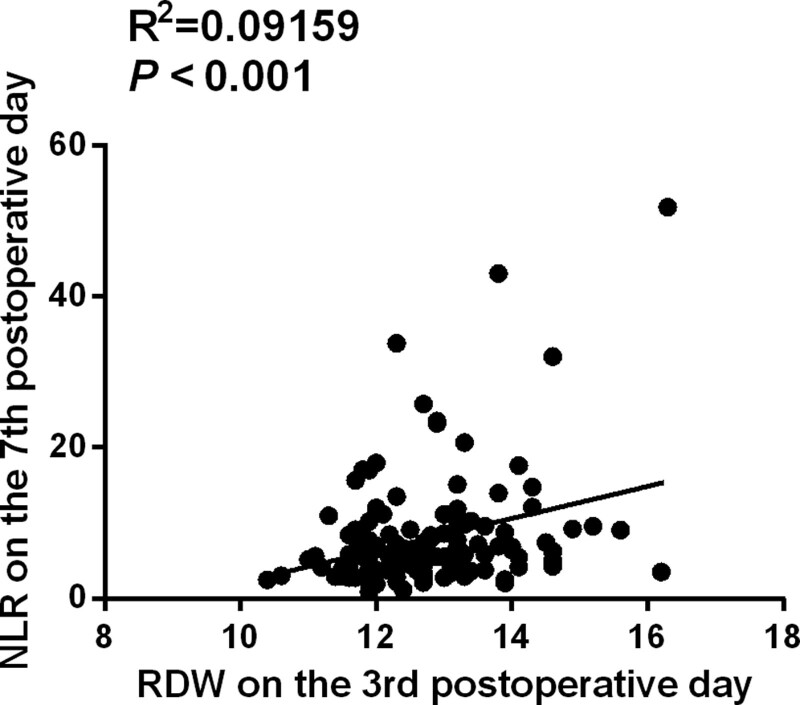
The correlation analysis between NLR and RDW. RDW on the 3rd postoperative day and NLR on the 7th postoperative day had a mild correlation (*R*^2^ = 0.09159, *P* < .001). NLR = neutrophil to lymphocyte ratio, RDW = red cell distribution width.

The results also showed that cutoff values of RDW and NLR for predicting the prognosis in aSAH patients were 13.05% and 6.97 respectively by ROC curves. The area under the curve of RDW on the 3rd postoperative day was 0.732 (95% CI: 0.633–0.831, *P* < .001), and the Youden index was 0.428. The sensitivity and specificity of RDW ≥ 13.05% on the 3rd postoperative day for predicting poor prognosis were 67.6% and 75.2% respectively (Fig. 3A). The area under the curve of NLR on the 7th postoperative day was 0.736 (95% CI: 0.631–0.841, *P* < .001), and the Youden index was 0.427. The sensitivity and specificity of NLR ≥ 6.97 on the 7th postoperative day for predicting poor prognosis were 41.2% and 89.9% (Fig. 3B). The sensitivity and specificity of the multivariate logistic model for predicting poor prognosis were 73.7% and 90% respectively (Fig. 3C). We also conducted a correlation analysis between NLR and RDW, and the result showed a mild correlation (*R*^2^ = 0.09159, *P* < .001) (Fig. 4).

## 4. Discussion

White blood cells and neutrophils were once considered sensitive biomarkers and proven to be associated with the prognosis of stroke. Tao C et al conducted a retrospective study exploring the associations between counts of blood cells and scores of mRS at 90 days in 336 patients with spontaneous cerebral hemorrhage. The results of multivariate analysis showed that higher white blood cells and neutrophils were associated with poor prognosis. Moreover, linear regression analysis showed that counts of these blood cells were significantly correlated with Glasgow Coma Scale score and cerebral hemorrhage volume at admission.^[12]^ Another study explored the associations of white blood cells at admission and cerebral hemorrhage dilation within 48 hours after onset, the result confirmed 207 (15.9%) cases with hematoma dilation. The higher white blood cells at admission, the lower risk of hematoma dilation. Hematoma dilation was negatively correlated with the count of neutrophils.^[13]^

NLR was the ratio of neutrophil to lymphocyte, combining the changes of the 2 blood cells, with more efficacy and accuracy compared to a single indicator for predicting inflammatory response. Sun Y. et al conducted one prospective study in 352 patients with acute cerebral hemorrhage. All patients were divided into different groups based on the NLR levels at admission to evaluate the associations of NLR with hematoma volume, disease severity, and prognosis. After a 3-month follow-up, patients with NLR ≥ 7.85 had higher NIHSS scores and more hematoma volumes, but not poor 3-month prognosis.^[14]^ In another study including 192 patients, there were 54 (28.1%) cases had neurological deterioration. NLR was independently correlated with neurological deterioration. NLR was the best variable for predicting poor outcomes.^[15]^ In our study, the associations of white blood cells, neutrophils, lymphocytes and NLR on different days with the 3-month prognosis were explored. The results of univariate analysis showed that counts of white blood cells and neutrophils on the preoperative, the 1st, the 3rd and the 7th postoperative days were all associated with prognosis. The results also showed that counts of lymphocytes on the 3rd and the 7th postoperative days, levels of NLR on the 1st, the 3rd and the 7th postoperative days were associated with prognosis. However, the result of multivariate logistic analysis only confirmed the association between NLR on the 7th postoperative day and the prognosis of aSAH patients.

NLR can get affected in systemic inflammatory response syndrome, sepsis, post-surgical state and many other confounders. The patients with abdominal infection had elevated counts of neutrophils before surgery and low lymphocyte counts. Critically ill patients with severe sepsis or septic shock had significantly highest values of neutrophil relative counts and marked lowest values of lymphocyte counts.^[16]^ Lymphocytes and neutrophils were found in the brain in areas of acute ischemic stroke. Post-stroke immunosuppression was also observed after stroke.^[17]^ NLR ≥ 5.9 was associated with pulmonary edema and high fever.^[18]^ Among patients with aSAH, whether a higher NLR level represents a severe or acute stress, or increased neutrophils and decreased lymphocytes originate from immunologic response to acute aSAH remains unknown. Delayed neuroinflammation or systemic inflammatory response syndrome after subarachnoid hemorrhage possibly resulted in a high NLR, which could be the trigger of secondary harmful hematologic or immunologic host responses. Neuroinflammation after acute cerebral hemorrhage could be reflected by acute leukocytosis and decline of lymphocytes. Infiltrated neutrophils could release inflammatory cytokines, and enhanced perifocal edema by promoting capillary permeability, cell swelling, and blood-brain barrier damage. Lymphocytes were the main immune regulatory cells, and their decrease and inactivation were markers of brain injury, which were the results of excessive activity of the sympathetic nervous system.^[19]^ On the other hand, patients with lower lymphocytes and higher neutrophils possibly developed concomitant infections,^[20]^ which also increase the risk of poor prognosis. Unfortunately, we did not pay attention to the characteristics of infected patients.

There were a few studies on the association of RDW with prognosis of aSAH patients. The results of one study including 274 patients with aSAH admitted to the intensive care unit confirmed a significant association between RDW levels and mortality.^[21]^ One systematic review and meta-analysis exploring the association of RDW with prognosis in aneurysmal subarachnoid hemorrhage patients confirmed that higher RDW was associated with a worse functional outcome, a worse discharge and 3-month functional outcome, and a higher in-hospital and 90-day mortality.^[22]^ In another study carried in 8175 community-dwelling adults 45 years or older and exploring the association between RDW and risk of death, the results showed that RDW was a widely available test and a strong predictor of mortality in the general population of adults 45 years or older.^[23]^ In our study, the univariate analysis results showed that the levels of RDW on the preoperative day, the 1st, 3rd and 7th postoperative days were all associated with the prognosis of aSAH patients, further suggesting that RDW might be a highly advantageous prognostic indicator. Moreover, the results of multivariate analysis confirmed the association between level of RDW on the 3rd postoperative day and the prognosis. The results by ROC curve in our study also showed that the cutoff value of RDW for predicting the prognosis in aSAH patients was 13.05%. The sensitivity and specificity of RDW ≥ 13.05% for predicting poor prognosis were 67.6% and 75.2%, both relatively high. Some possible mechanisms by which higher RDW was associated with poor prognosis in aSAH patients were explored. Higher RDW levels were associated with endothelial dysfunction and increased levels of inflammatory cytokines, and reflected the potential pro-inflammatory state.^[24,25]^ The higher the RDW level, the greater the likelihood of thrombosis formation in patients. The increase of RDW level indicated the increase of immature reticulocytes, which had worse deformability and stronger thrombolytic ability than mature erythrocytes.^[26]^ Deformed erythrocytes could accelerate the process of platelet aggregation, damage blood vessels, and reduce blood flow.^[27]^ Higher RDW levels were also associated with lower antioxidant levels and higher oxidative stress levels,^[28]^ which could weaken the deformability of erythrocytes, increase adhesion between erythrocytes and endothelial cells, and promote thrombosis.^[7]^ In addition, the lifespan of erythrocytes was approximately 120 days. Therefore, RDW possibly reflect the long-term inflammatory state of aSAH patients.

The prognosis differences among patients with different quartiles of RDW and NLR in Table 3 confirmed that the proportions of cases with higher quartiles of RDW or NLR were higher than lower quartiles in patients with poor prognosis, further suggesting NLR and RDW might be more superior indicators than other blood cells for predicting prognosis in aSAH patients. We also showed a mild correlation between NLR and RDW. Perhaps there is some inherent connection between NLR and RDW, and exploring the possible connection mechanism between NLR and RDW is our future research direction.

Our study have some limitations. First, the dataset had some bias. There were two-thirds or over cases with a good Hunt-Hess grade (I/II) in all patients or in patients with good outcome, one-third or less cases with a poor Hunt-Hess grade (III/IV/V). The good outcome was possibly driven in general by a favorable natural history due to minor SAH in the majority of patients with a good Hunt-Hess grade. Second, the patients with good outcome by far outweighed the patients with poor outcome, so the patients with poor outcome might be underpowered. Third, the number of patients was not large enough, which possibly caused some bias. We will investigate the associations in larger-sample study. Finally, characteriazatioin of patients who developed hospital or out-hospital infections did not get noticed in our manuscript, which was an additional limitation.

## 5. Conclusion

In summary, higher RDW on the 3rd postoperative day and NLR on the 7th postoperative day were possibly associated with poor prognosis of aSAH patients. We should pay attention to the RDW and NLR levels during different hospitalization periods, especially in the short postoperative period. Moreover, the cutoff values for predicting prognosis need to be validated in larger-sample studies.

## Acknowledgments

We are thankful to all the medical staff in the Neurointensive care unit and the statistician, in The First Affiliated Hospital of Yangtze University.

## Author contributions

**Conceptualization:** Jie Min, Yongfeng Zhao, Xian Wang.

**Data curation:** Xian Wang.

**Formal analysis:** Yongfeng Zhao.

**Methodology:** Jie Min, Jian Zhao.

**Project administration:** Xian Wang, Jian Zhao.

**Writing – original draft:** Jie Min, Yongfeng Zhao.

**Writing – review & editing:** Jie Min, Xian Wang.
